# Motor imagery reinforces brain compensation of reach-to-grasp movement after cervical spinal cord injury

**DOI:** 10.3389/fnbeh.2015.00234

**Published:** 2015-09-11

**Authors:** Sébastien Mateo, Franck Di Rienzo, Vance Bergeron, Aymeric Guillot, Christian Collet, Gilles Rode

**Affiliations:** ^1^ImpAct Team, Lyon Neuroscience Research Center, Université Lyon 1, Université de Lyon, INSERM U1028, CNRS UMR5292Lyon, France; ^2^Hospices Civils de Lyon, Hôpital Henry Gabrielle, Plateforme Mouvement et HandicapLyon, France; ^3^Centre de Recherche et d’Innovation sur le Sport, EA 647, Performance Motrice, Mentale et du Matériel, Université Lyon 1, Université de LyonVilleurbanne, France; ^4^Ecole Normale Supérieure de Lyon, CNRS UMR5672Lyon, France; ^5^Institut Universitaire de FranceParis, France

**Keywords:** tetraplegia, motor imagery, grasping, brain plasticity, motor control, kinematic, recovery, compensation

## Abstract

Individuals with cervical spinal cord injury (SCI) that causes tetraplegia are challenged with dramatic sensorimotor deficits. However, certain rehabilitation techniques may significantly enhance their autonomy by restoring reach-to-grasp movements. Among others, evidence of motor imagery (MI) benefits for neurological rehabilitation of upper limb movements is growing. This literature review addresses MI effectiveness during reach-to-grasp rehabilitation after tetraplegia. Among articles from MEDLINE published between 1966 and 2015, we selected ten studies including 34 participants with C4 to C7 tetraplegia and 22 healthy controls published during the last 15 years. We found that MI of possible non-paralyzed movements improved reach-to-grasp performance by: (i) increasing both tenodesis grasp capabilities and muscle strength; (ii) decreasing movement time (MT), and trajectory variability; and (iii) reducing the abnormally increased brain activity. MI can also strengthen motor commands by potentiating recruitment and synchronization of motoneurons, which leads to improved recovery. These improvements reflect brain adaptations induced by MI. Furthermore, MI can be used to control brain-computer interfaces (BCI) that successfully restore grasp capabilities. These results highlight the growing interest for MI and its potential to recover functional grasping in individuals with tetraplegia, and motivate the need for further studies to substantiate it.

## Introduction

Individuals with tetraplegia are challenged with dramatic sensorimotor deficits caused by cervical spinal cord injury (SCI). Active grasp is lost due to hand and finger muscle paralysis although compensation is possible (Long and Lawton, 1955; Kirshblum et al., 2007). Compensations are restricted to bimanual grasp after C5 SCI while other grips using the mouth or tongue compensate for grasp after C4 SCI. Tenodesis grasp relies on the spared wrist extensor muscle after C6 and C7 SCI. Indeed, tendon shortening of either the *flexor digitorum* or *flexor pollicis longus* occurs simultaneously to wrist extension resulting respectively in passive palmar or lateral grip (Mateo et al., 2013). These upper limb movement modifications are accompanied by increased activity of contralateral sensorimotor cortex, supplementary motor area and ipsilateral cerebellum, varying according to the SCI level (Bruehlmeier et al., 1998; Curt et al., 2002; Cramer et al., 2005; Jurkiewicz et al., 2007; Kokotilo et al., 2009). Improving grasping abilities are important issues for recovering autonomy of individuals with tetraplegia (Long and Lawton, 1955; Beninato et al., 2004). Consequently, rehabilitation aims to restore reach-to-grasp using physical and occupational therapies (Woolsey, 1985; Kirshblum et al., 2007).

There is growing evidence of motor imagery (MI) benefits for neurological rehabilitation of upper limb movements (Warner and McNeill, 1988; Jackson et al., 2001). The mental representation of an action without any physical execution engages brain motor regions overlapping those activated by physical practice (PP; Decety and Grèzes, 1999; Pfurtscheller, 2001). This functional equivalence principle was early described in healthy individuals (Jeannerod, 1994; Lotze and Halsband, 2006; Hanakawa et al., 2008; Munzert et al., 2009) and in individuals with SCI (Decety and Boisson, 1990; Lotze and Halsband, 2006; Di Rienzo et al., 2014a). Thus MI enables active stimulation of brain motor areas promoting brain plasticity (Lotze and Halsband, 2006; Dunlop, 2008) associated with positive effects on motor performance (Driskell et al., 1994).

Thereby, MI could constitute a promising approach to rehabilitate grasping abilities after C6 and C7 tetraplegia. Furthermore, individuals with C4 and C5 tetraplegia could imagine movements to control a device that can replace grasping using brain-computer interfaces (BCI; Pfurtscheller et al., 2003a). BCI extract the somato-topically organized sensorimotor rhythms from brain activity during MI (Yuan and He, 2014). The BCI then transforms brain activity into signals driving an output to control a grasping device. A BCI requires several steps including: (i) preprocessing to improve signal-to-noise ratio; (ii) frequency selection where the greatest amplitude of sensorimotor rhythms during MI are measured; and (iii) detection and classification where participants are extensively trained to imagine a movement with or without cues, which results in a less adaptive synchronous BCI (cue-paced) or a more adaptive asynchronous BCI (self-paced).

The aim of this literature review is to address the effectiveness of MI upon upper limb rehabilitation after tetraplegia. More precisely, we will investigate behavioral changes (reduction of upper limb functional deficit) and brain activity changes in response to MI intervention. Understanding the potential for MI to improve motor performance by reinforcing compensations or potentiating recovery, with or without influence on brain plasticity is of particular interest.

## Materials and Methods

We selected full articles from the U.S. National Library of Medicine® (MEDLINE) between 1966 and June 2015 assessing the effect of MI intervention in individuals with complete motor tetraplegia. Included are single case, case series and control case studies of MI intervention on upper limb and tongue trials with pre-post movement performance or brain activity recordings. Excluded studies are those without grasping deficit e.g., in individuals with paraplegia, without complete SCI, and/or when MI intervention only involved lower limb movements. We analyzed behavioral improvement due to MI intervention on several dependent variables (performance, velocity, manual dexterity and kinematics) while also considering brain activity changes in response to MI.

## Results

### Studies

**Figure 1:**
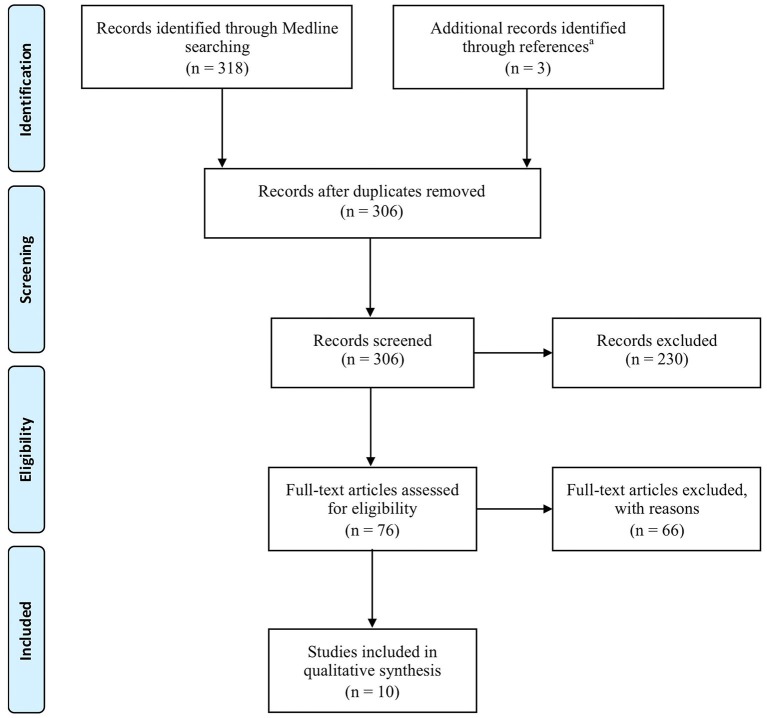
**Flow diagram of review process according to the Preferred Reporting Items for Systematic Reviews and Meta-Analyses (PRISMA—Moher et al., 2009)**. ^a^The three identified papers were (Laffont et al., 2000; Hoffmann et al., 2002; Collinger et al., 2013b).

Figure 1 provides a flowchart that illustrates and summarizes the literature review process we used. From the 306 articles screened, papers that did not fulfill at least one of our exclusion criteria were not considered. This resulted in exclusion of 230 articles after reading the title and/or abstract. Among the 76 remaining full-text articles, 66 papers were rejected for the following reasons:

1. MI studies with no tetraplegic participants (Boschker et al., 2000; Pfurtscheller et al., 2003b; Wilson, 2003; Erfani and Erfanian, 2004; Erfanian and Erfani, 2004; Grush, 2004; Grosjean et al., 2007; Szpunar et al., 2007; Miller et al., 2010; Müller-Putz et al., 2010; Olsson and Nyberg, 2010; Schill et al., 2011; Viswanathan et al., 2012; Papageorgiou et al., 2013; Smits-Engelsman and Wilson, 2013; Kondo et al., 2014; Grosprêtre et al., 2015; Malik et al., 2015);
2. Studies including tetraplegic participants with no MI intervention (Saxena et al., 1995; de Castro and Cliquet, 2000a,b; Laffont et al., 2000, 2007, 2009; Memberg and Crago, 2000; Thorsen et al., 2001, 2014; Hoffmann et al., 2002; Nunome et al., 2002; Taylor et al., 2002; Remy-Neris et al., 2003; Shimada et al., 2003; Cornwall and Hausman, 2004; Pfurtscheller et al., 2005; Anderson et al., 2008; Robinson et al., 2010; de los Reyes-Guzmán et al., 2010; Martin et al., 2012; Siedziewski et al., 2012; Coignard et al., 2013; Collinger et al., 2013a,b; Cortes et al., 2013; Mateo et al., 2013, 2015a; Wodlinger et al., 2015);
3. MI of lower limb movements only (Pfurtscheller et al., 2008; Flanagin et al., 2009; Tcheang et al., 2011);
4. Articles without pre-post measures (Decety and Boisson, 1990; Lacourse, 1999; An et al., 2006; De Mauro et al., 2011; Ajiboye et al., 2012; Blokland et al., 2012, 2014; Grangeon et al., 2012b; López-Larraz et al., 2012; Fiori et al., 2014; Müller-Putz et al., 2014); and
5. Articles without movement performance assessment (Enzinger et al., 2008; Di Rienzo et al., 2014b, 2015; Faller et al., 2014; Scherer et al., 2015; Tidoni et al., 2015).

We thus included 10 studies involving five single case (Pfurtscheller et al., 2000; Müller-Putz et al., 2005; Grangeon et al., 2010, 2012a; Rohm et al., 2013), two case series (Onose et al., 2012; Vučković et al., 2015) and three control cases (Cramer et al., 2007; Di Rienzo et al., 2014c; Mateo et al., 2015b). We scored the quality of these studies using the Single-Case Experimental Design (SCED) scale (Tate et al., 2008), the 3 min critical appraisal for case series (Chan and Bhandari, 2011) and the Physiotherapy Evidence Database (PEDro) scale (Maher et al., 2003; de Morton, 2009). The SCED scores were 5/10 (Grangeon et al., 2010, 2012a) and 3/10 (Pfurtscheller et al., 2000; Müller-Putz et al., 2005; Rohm et al., 2013). The absence of a baseline and statistical analysis explained the difference in score. Similarly, the control case series studies all had a 5/10 PEDro score. We note that PEDro scores below 6/10, have been considered as low quality (Paci et al., 2010). Only the two case series studies were evaluated as having so-called high quality (Chan and Bhandari, 2011).

### Participants

The 10 studies involved a total of 34 participants with tetraplegia and 22 healthy age-matched controls. Mean age was 33 years (22–42). SCI Levels were C4 (*n* = 3), C5 (*n* = 9), C6 (*n* = 16) and C7 (*n* = 6; see Table 1). All participants were at a chronic stage (mean = 31 months after SCI 3.5–84) with the exception of two who were included 3 and 4 months after SCI (Vučković et al., 2015). Furthermore, all studies included participants with complete motor lesion AIS A or B, except the article by Onose et al. (2012), which included two participants with AIS C. MI vividness was on average self-rated at 3.6/5 (from 3.3–4.1) and 3.3/5 (from 2.2–3.8) for visual and kinesthetic MI modalities, respectively (Grangeon et al., 2012a; Di Rienzo et al., 2014c; Mateo et al., 2015b; Vučković et al., 2015).

**Table 1:** **Participants and studies characteristics**.

							MI				Outcome	
Reference	Study	SCI level	Patient	AIS Score (A–E)	Mean age (years)	Mean delay (months)	Training before MI	Sessions	Duration (min)	Content	Behavioral activity	Brain
Pfurtscheller et al. (2000)	SC	C5	1	NA	22	24	No	NA	NA	Hand foot	Grasping*	EEG
Müller-Putz et al. (2005)	SC	C5	1	A	42	84	No	3	540	Hand foot	Grasping*	EEG
Cramer et al. (2007)	CC	C5	5	A, B	30 *(SD 4)*	Up to 12	Observation^c^	7	420	Tongue foot	Movement frequency	fMRI
		C6	1^a^
Grangeon et al. (2010)	SC	C6	1	A	41	32	PP^d^	20	300	Reaching grasping	Strength reaching**	NA
Grangeon et al. (2012a)	SC	C6	1	A	23	8	PP	15	675	Reaching grasping	Dexterity*** Grasping**	NA
Onose et al. (2012)	CS	C4	2	A, B, C	33 *(23–51)*	66 *(6–202)*	Observation^c^	5	900	Hand ankle	Grasping*	EEG
		C6	3
		C7	4
Rohm et al. (2013)	SC	C4	1	A	42	48	No	43	NA	Hand	Grasping*	EEG
Di Rienzo et al. (2015)^e^	CC	C6	5	A, B	30 *(18–40)*	14 *(6–30)*	PP	15	675	Reaching grasping	Movement duration	MEG
		C7	1^b^
Vučković et al. (2015)	CS	C5	2	A, B	39 *(32–45)*	3.5 *(3–4)*	No	4–10	NA	Hand	Grasping*	EEG
Mateo et al. (2015b)^e^	CC	C6	5	A, B	30 *(18–40)*	14 *(6–30)*	PP	15	675	Reaching	Grasping**	MEG
		C7	1							grasping		

Abbreviations: NA, Not Available; SC, single case; CS, case series; CC, control case; C, cervical level of SCI; MI, Motor Imagery; PP, Physical Practice; EEG, Electroencephalography; fMRI, functional Magnetic Resonance Imaging; MEG, Magnetoencephalography. Number of control participants included was ^a^10, ^b.^6, ^c^observation of video showing the movement to perform, ^d^crossover design with PP before MI and after, ^e^Both references are from the same scientific team and included the same group of participants with tetraplegia. *Grasping was achieved with MI based BCI controlling a grasping device, **kinematic of a reaching or reach-to-grasp task, ***dexterity was assessed using the Block and Box Test and the Minnesota Manual Dexterity Test.

### Outcome Measures

MI intervention effects were assessed through clinical and kinematic outcomes, along with changes in brain activity. These include: (i) passive range of motion measured with a goniometer (Grangeon et al., 2010); (ii) muscle strength assessed by the Manual Muscle Test (Grangeon et al., 2010; Vučković et al., 2015); (iii) manual dexterity outcome using the Minnesota Manual Dexterity Test (MMDT), the Block and Box Test (BBT; Grangeon et al., 2012a) and the Grasp and Release Test (GRT; Müller-Putz et al., 2005); and (iv) kinematic outcomes during reaching and reach-to-grasp movements including temporal parameters e.g., movement time (MT) and spatial parameters e.g., trajectory, joint motion, wrist extension angle during grasping (Grangeon et al., 2010, 2012a; Mateo et al., 2015b). In addition, outcomes of grasping effectiveness have also been done using a BCI device controlled by MI (Pfurtscheller et al., 2000; Müller-Putz et al., 2005; Onose et al., 2012; Rohm et al., 2013; Vučković et al., 2015). Finally, 8 studies investigated brain activity changes in response to MI using electroencephalography (EEG; Pfurtscheller et al., 2000; Müller-Putz et al., 2005; Onose et al., 2012; Rohm et al., 2013; Vučković et al., 2015), functional magnetic resonance imaging (fMRI; Cramer et al., 2007) or magnetoencephalagraphy (MEG; Di Rienzo et al., 2014c; Mateo et al., 2015b).

### MI Interventions

Mean data showed that participants rehearsed mentally during 598 min (range from 300–900). However, one study did not report MI practice duration (Rohm et al., 2013; Table 1). Instead, Rohm et al. (2013) indicated that participants performed 413 MI trials. The mean number of MI sessions was 14 (range from 3–43) over 10 weeks (range from 0.4–52). Practice before MI consisted of video observation (Cramer et al., 2007; Onose et al., 2012) or PP with a crossover design (Grangeon et al., 2010) and without crossover (Grangeon et al., 2012a; Di Rienzo et al., 2014c; Mateo et al., 2015b). Conversely, there was no practice before MI in the other studies (Pfurtscheller et al., 2000; Müller-Putz et al., 2005; Cramer et al., 2007; Rohm et al., 2013; Vučković et al., 2015). SCI participants imagined single-joint movements of: (i) wrist flexion/extension (Di Rienzo et al., 2014c; Mateo et al., 2015b); (ii) hand movements (Müller-Putz et al., 2005; Rohm et al., 2013); (iii) arrhythmic flexion/extension of both finger and ankle (Onose et al., 2012); or (iv) functional movement of reaching and reach-to-grasp (Grangeon et al., 2010, 2012a; Di Rienzo et al., 2014a; Mateo et al., 2015b).

In cases of C4-C5 SCI, grasping was achieved using MI based BCI via an EEG to control a motorized hand orthosis (Pfurtscheller et al., 2000), an implanted functional electrical stimulation (FES; Müller-Putz et al., 2005), a surface FES (Rohm et al., 2013; Vučković et al., 2015) or a grasping robot (Onose et al., 2012). The EEG recorded the electrical activity over the sensorimotor cortex (electrodes were located at C3, Cz and C4 according to the 10–20 international system). Then, the frequency range showing the highest sensorimotor rhythms within the alpha/mu and beta bands (8–13, 13–35 Hz) were tailored to each participant. All but one study used two imagined movements to generate the output signal and control the device, with the exception of Müller-Putz et al. (2005) who only used one imagined movement to control the device. The total amount of MI training ranged between 3 and 1012 sessions (see Table 2). The ratio between correctly classified trials and the total number of trials (i.e., the classification accuracy; Graimann et al., 2010) ranged between 71 and 95%. Finally, SCI participants controlled the device to restore grasping using either self-paced MI i.e., asynchronous BCI (Müller-Putz et al., 2005; Vučković et al., 2015) or cue-paced MI i.e., synchronous BCI (Pfurtscheller et al., 2000; Onose et al., 2012; Vučković et al., 2015).

**Table 2:** **MI Based BCI efficacy studies to compensate grasping function**.

Reference	Patient	BCI type	Frequency (Hz)	MI Detection (cue)	Class	MI	Classifier	Maximum of sessions	Classification accuracy^1^	Device controlled	BCI output
Pfurtscheller et al. (2000)	1	S	15–18	Auditory	2	R, F	LDA^2^	NA	95%^3^	Orthosis	F: orthosis opening
											R: orthosis closing
Müller-Putz et al. (2005)	1	A	14–16 18–22	No	1	L, F	LDA^2^	3	73%	I FES^4^	L: FES sequence start^5^
Onose et al. (2012)	9	S	13–30	Visual	2	L, R, F, N	LDA^2^	10	71%	Robot^6^	Two classes for robot start and stop^7^
Rohm et al. (2013)	1	S	23–26	Visual	2	R, F	LDA^2^	119	71%	S FES^8^	R: BCI switch^9^
Vučković et al. (2015)	2	S	4–8	Visual	2	R, F	NA	1012	85%	S FES^10^	R: FES start
			12–16								E: FES stop

*Abbreviations: BCI, Brain-computer interface; MI, Motor Imagery; S, synchronous; A, Asynchronous; R, Right hand; L, Left hand; E, Eye closing; F, Feet; N, No movement i.e., relaxation; NA, Not Available; I, Implanted; S, Surface; FES, Functional Electrical Stimulation. ^1^Is a BCI measure of performance given by a ratio of the number of correctly classified trials (successful attempts to perform the required MI) and the total number of trials (Graimann et al., 2010), ^2^Linear Discriminant Analysis is a linear classifier categorizing MI in several classes that can be affected to different function, ^3^Accuracy was 65% when participant imagined L vs. R, improved to 75% when he imagined movement of L/R or F vs. N and finally improved to 95% when he imagined R vs. F, ^4^The implanted FES was the freehand® system, ^5^The three-part sequence of FES consisted in (i) finger extension (including thumb), (ii) finger flexion triggering palmar grip and (iii) thumb flexion triggering lateral grip, ^6^Upper limb robot with 10 degrees of freedom, ^7^Depending on participants, the pair for robot start stop was respectively L/N (*n* = 2), L/F (*n* = 3), F/N (*n* = 1), L/R (*n* = 4), ^8^Surface FES restored elbow flexion and grasping with palmar grip, ^9^Four BCI switch producing the sequence of (i) elbow extension then, (ii) grasping then, (iii) elbow flexion to eat then, (iv) elbow extension and hand opening to release the object, ^10^Surface FES elicited stimulation of the wrist extensor carpi radialis longus using stimulation parameters set with pulse of 300 μs duration, 15 mA amplitude and 30 Hz frequency*.

### Clinical Evidence of MI Effectiveness

Using a crossover design, Grangeon et al. (2010) reported motor improvement whatever the order of practice (PP before MI or after). The chronic C6 SCI participants exhibited; (i) increased amplitude of passive elbow flexion (from 90° to 145°), and (ii) increase in strength of both the elbow flexor and extensor muscles respectively from 2 to 4/5 and 1 to 4/5 on the Manual Muscle Testing score, indicating that the movement could subsequently be performed against gravity and even against a light resistance after MI training. Similarly, after training of triggered electrical stimulation using MI-based BCI, Vučković et al. (2015) showed that one of the two C5 acute SCI participants increased *brachioradialis* strength (from 1 to 3/5, i.e., the initial palpable muscle contraction changed to full elbow flexion range of motion against gravity).

In response to MI of possible upper limb movements (e.g., grasping), one C6 SCI participant demonstrated increased manual dexterity as shown by; (i) significant improvement in the BBT; and (ii) decreased time to complete the MMDT (Grangeon et al., 2012a). Similarly, six C6-C7 SCI participants showed decreased variability of MTs during a complete reach-to-grasp sequence, including bringing an apple to the mouth and then putting it back in its initial location (Di Rienzo et al., 2014c). In addition, after learning a movement sequence using MI of either the right foot or the tongue, seven C5 to C7 SCI participants only exhibited a decreased in MT to complete the sequence with the tongue (i.e., during practice of possible movements; Cramer et al., 2007).

Furthermore, training of MI based BCI resulted in compensation of grasping movements with the successful control of the BCI device. By controlling surface FES, one C4 SCI participant showed decreased MT when grasping, along with writing his own name or eating an ice cream cone (Rohm et al., 2013). Similarly, one C5 SCI participant successfully grasped a paperweight in the GRT and moved it five times from one place to another (Müller-Putz et al., 2005). By controlling a motorized hand orthosis, another C5 SCI participant grasped and ate an apple (Pfurtscheller et al., 2000). In addition, by controlling an upper limb robot 3 of 9 C5 SCI participants successfully grasped a glass and drank from it (Onose et al., 2012; Table 2).

### Kinematic Evidence of MI Effectiveness

**Figure 2:**
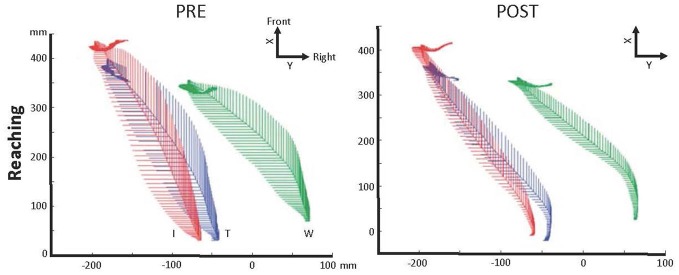
**Illustration of the motor control improvement after motor imagery (MI) practice in one C6 SCI participant**. Kinematic recordings showing trajectory variability decrease of the right index finger (I—red), thumb (T—blue) and wrist (W—green) during reaching in the contralateral space immediately after MI practice, 1 and 3 months later (POST; adapted with permission from Grangeon et al., 2012a). Abbreviations: X, X-axis sets in participant’s frontal plane; Y, Y-axis sets in participant’s sagittal plane.

Variability of hand trajectory decreased during reaching toward a central target placed 15 cm from a starting point in one C6 SCI participant (Grangeon et al., 2010). Similarly, variability of hand trajectory decreased by 58% during reach-to-grasp of a glass placed 40 cm in the front of the C6 SCI participant (Grangeon et al., 2012a; Figure 2). In addition, MT decreased by about 29% (Grangeon et al., 2012a). Finally, six C6-C7 SCI participants increased their wrist extension angle by 28% (i.e., wrist extension triggering tenodesis grasp) during reach-to-grasp of an apple placed at 35 cm (Mateo et al., 2015b). Motor improvements were preserved during retention tests, up to 2 months (Mateo et al., 2015b) and 3 months (Grangeon et al., 2012a) after MI training was stopped.

### Brain Activity Modification in Response to MI

**Figure 3:**
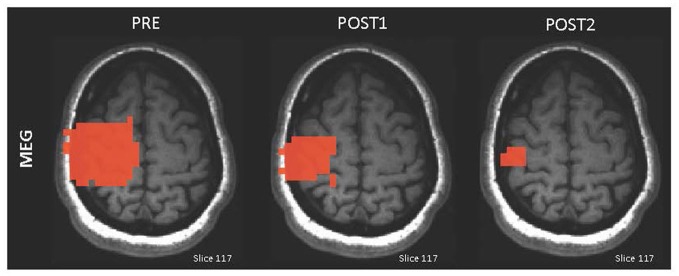
**Illustration of the adaptive brain plasticity after MI practice in one C6 SCI participant**. Magnetoencephalography (MEG) maps displaying the contralateral sensorimotor activation decrease immediately after MI training (POST1) and 2 months later (POST2; adapted with permission from Mateo et al., 2015a).

In response to MI of impossible paralyzed movements (e.g., foot), seven C5 to C7 SCI participants showed increased activation in the left putamen and globus pallidus during imagined foot movements measured by fMRI (Cramer et al., 2007). Similarly, one C5 SCI participant performing foot-movement MI exhibited increased amplitude of EEG sensorimotor rhythms in the cortical areas controlling the foot (Pfurtscheller et al., 2000). Conversely, MI practice of possible movements spared from SCI (e.g., reach-to-grasp) resulted in a decrease in the left premotor cortex activity during complete reach-to-grasp with the right hand in six C6-C7 SCI participants measured by MEG (Di Rienzo et al., 2014c). Similarly, six C6-C7 SCI participants exhibited decreased contralateral sensorimotor activity measured by MEG during wrist-extension triggering of the tenodesis grasp (Mateo et al., 2015b; Figure 3).

## Discussion

The interest of using MI practice during upper limb rehabilitation after tetraplegia is growing. The effectiveness of MI to promote upper limb rehabilitation after tetraplegia remains nevertheless poorly understood. The aim of this review is to address the extent to which MI practice of possible movements spared from cervical SCI or impossible paralyzed movements may activate and reinforce cerebral networks in order to promote recovery or reinforce compensation during rehabilitation of reach-to-grasp movement after tetraplegia.

The training effects of MI on possible movement recovery have been studied through strength assessments using the Manual Muscle Test (Compston, 2010). Indeed, one chronic C6 SCI participant underwent surgical tendon transfer of the *biceps brachii* onto that of the *triceps brachii* and exhibited strength increase in both elbow flexor and extensor muscles in response to 2 weeks of MI practice (Grangeon et al., 2010). Here, improvement in strength relies on central modifications favoring: (i) change in *biceps brachii* function from elbow flexion to extension; and (ii) compensation of the loss of the transferred *biceps brachii* by the two remaining elbow flexor muscles (*brachioradialis and brachialis*). Moreover, strength increase was reported in the *brachioradialis* of one acute C5 SCI participant after seven MI sessions of grip preparation aimed at restoring grasp using surface FES controlled by BCI (Vučković et al., 2015). This is consistent with a similar strength increase in the little finger abductor and elbow flexor muscles reported in response to MI in healthy individuals (Yue and Cole, 1992; Ranganathan et al., 2004). Associated with these gains, EEG showed that the amplitude of sensorimotor rhythms increase during maximal voluntary contraction of the trained muscles, particularly during the power signal decrease i.e., the event related desynchronization (Ranganathan et al., 2004). From these observations, gain in strength has been attributed to central motor planning improvement, such as better recruiting and synchronizing of motoneurons in absence of muscle hypertrophy (Yue and Cole, 1992). Furthermore, based on EEG results, Ranganathan et al. (2004) concluded that MI “*enhances the cortical output signal, which drives the muscles to higher activation levels and increases strength*”. Although these results should be associated with processes of natural recovery and rehabilitation, MI may have the potential to strengthen motor commands of upper limb movements, thus improving recovery.

Results from the other studies suggest a potential MI effect on compensation improvements during reach-to-grasp. One example is the BBT and MMDT improvements in response to 675 min of upper limb MI in a complete C6 SCI participant (Grangeon et al., 2012a). This may be related to the kinematic measures that reveal wrist extension increases, in the tenodesis grasp of six C6-C7 SCI participants, also after 675 min of MI practice (Mateo et al., 2015b). Taken together, the results suggest that hand dexterity improved which can be explained by endpoint movement accuracy and reinforcement of the tenodesis grasp. Thus, MI may have strengthened the motor planning (Mateo et al., 2015a). Furthermore, the reduction in hand trajectory variability indicates a reduction in both reaching and grasping movement inefficiencies (Grangeon et al., 2010, 2012a). Since reach-to-grasp is sub-divided into a transport phase (specifically tested by reaching) and a grasping phase (Jeannerod, 1984), overall motor control improvements involve both phases. This suggests that MI also reinforces the motor planning based on the kinematic invariant of minimal cost (Mateo et al., 2015a). Additionally, movement duration is also an index of performance. In response to MI of possible movements, duration of both reach-to-grasp and tongue sequence movements decrease (Cramer et al., 2007; Grangeon et al., 2012a) along with movement duration variability (Di Rienzo et al., 2014c). Hence, MI of possible movements is likely to: (i) promote the learning of new movement sequences; and (ii) improve the tenodesis grasp strategy that is one cause of MT reduction after tetraplegia (Mateo et al., 2015a). Therefore, these results imply that MI of possible movements reinforces strategies of movement planning according to kinematic invariants like minimal cost and endpoint movement accuracy (Mateo et al., 2015a). Here again, the effects of MI are thought to be limited to the central level by reinforcing the necessary motor commands and by building new motor commands through brain plasticity (Dunlop, 2008).

MI can induce brain plasticity through active stimulation of brain motor networks (Lotze and Halsband, 2006; Dunlop, 2008). Consequently, MI has been used to test if it can reduce the abnormally increased brain activity after tetraplegia (Kokotilo et al., 2009) using both impossible movements (e.g., foot) or possible movements (e.g., hand). After 420 min of MI training based on impossible foot movement sequences, Cramer et al. (2007) reported increased activity in the left putamen and globus pallidus. These areas are associated with motor learning and foot movements and can thus be considered as new movements that are not physically practiced due to paralysis. Consequently, this change in brain activity may relate to the first stage of motor learning (Karni et al., 1998). However, the absence of brain activity reduction in response to MI of impossible movement could not be definitively concluded because MI practice duration was short (7 days) and further practice could have resulted in the hypothesized brain activity changes (Doyon and Benali, 2005). Conversely, after 675 min of MI on possible upper limb movements, the additional recruitment in premotor cortex during grasping, compared to healthy control participants before MI training, was no longer observed (Di Rienzo et al., 2014c). In addition, the abnormally increased activity within the contralateral sensorimotor cortex during wrist-extension, was reduced and matched with healthy controls (Mateo et al., 2015b). Since both premotor- and sensorimotor cortex have been associated with motor planning during MI (Guillot et al., 2014), reduced activity could be due to “automation” thus involving cortical motor regions, parietal cortex, basal ganglia, and cerebellum (Doyon and Benali, 2005; Doyon et al., 2009; Vahdat et al., 2015). Along these lines, Cramer et al. (2007) reported that movement automation was associated with increased activity in basal ganglia even if C6 SCI participants performed MI of impossible foot movements. There is no additional evidence of brain activity changes within sub-cortical and cerebellar areas, related to MI learning after tetraplegia. However, considering functional equivalence between MI and PP, brain plasticity could be inferred from motor learning through actual practice. Hence, healthy participants exhibited activity decrease in the motor related brain areas involving cortico-basal ganglia and cortico-cerebellar pathways associated with more efficient skills requiring less energy (Doyon et al., 2009). Vahdat et al. (2015) recently investigated brain-spinal cord activity changes after actual training of finger movements. Healthy individuals showed that connectivity between sensorimotor cortex and the spinal cord decreased while that between cerebellum and the spinal cord was reinforced during learning. Whether these changes are less likely to occur after MI due to motor command inhibition remains unknown. Nevertheless, spinal cord plasticity induced by MI practice cannot be excluded since inhibition is weakened after SCI (Roy et al., 2011; Di Rienzo et al., 2014b) while corticospinal facilitation below motor threshold can occur (Stinear, 2010). Consequently, further studies should look for plasticity evolution in the motor related brain areas even considering the spinal cord after MI practice. Finally, the results we reviewed here, generally suggest that MI practice of possible and impossible movements resulted in a fundamental difference in brain plasticity. MI practice of impossible movements could be seen as learning a new task due to paralysis. Conversely, there is some evidence that increased activity caused by SCI is negated after MI training of possible movements. It is also noteworthy to mention that cortical changes, in particular after MI training of possible movements, could be associated with motor control and movement performance improvement due to the reinforcement of compensatory movement (e.g., tenodesis grasp).

Although there have been limited studies, promising evidence of MI based BCI efficacy to compensate for inability to grasp is also accumulating. Indeed, participants with C4 and C5 tetraplegia have gradually become able to control a grasping BCI device using extensive MI training of impossible movements (e.g., right, left hand or feet). In parallel, sensorimotor rhythms of imagined foot movements matched those from healthy control participants after 5 months of training (Pfurtscheller et al., 2000). This indicates that MI of impossible movements could restore brain activity reversing the reduction of sensorimotor rhythms which was previously reported during MI of impossible movements (Lacourse, 1999). As in healthy populations, MI has the ability to reinforce brain activity, leading to its use in controlling a BCI device. Nevertheless, the diversity of devices (e.g., surface or implanted FES, motorized hand orthosis or grasping robot) and methods (based on choice of frequency recorded or on type of movement imagined) or data processing (EEG data treatment leading to device control output) require further development to promote their routine use in rehabilitation. In particular, several issues should be further addressed e.g., the limited number of degrees of freedom controlled by MI based BCI, along with the reduction of MI training duration to control the device, from 5 months to 3 days respectively in the articles by Pfurtscheller et al. (2000) and Müller-Putz et al. (2005).

## Conclusion

This literature review included 10 studies involving MI training for cervical SCI published over the last 15 years. The interest for using MI stems from its use as a complementary technique during grasping rehabilitation after tetraplegia. The results we briefly described here show motor control and performance improvement in response to MI of possible movements in individuals with SCI. This could be attributed to the improvement of compensation movements like the tenodesis grasp and to a lesser extend strength recovery. In addition, thus far it appears that only MI of possible movements can reduce abnormally increased brain activity as compared to control participants. Taken together, motor performance and brain plasticity reflect functional and structural changes in the central nervous system enabling the improvement of the compensated grasping movements. Furthermore, MI based BCI is a promising procedure which could further complete rehabilitation programs, in particular for the case of high level SCI (C4 and C5). Despite promising results and potential use of MI in rehabilitation methods, current studies provide only a weak level of evidence (Guyatt et al., 2008). Thus at this point, any generalization of results must be taken with caution and future studies should strive to eliminate potential bias due to low quality, and small sample sizes of SCI participants. Further investigations providing randomized controlled trials with a high evidence level are needed to confirm the MI effects for grasp rehabilitation after tetraplegia and to elucidate any changes in brain plasticity.

## Author Contributions

SM, CC, GR made substantial contributions to the conception, acquisition, analysis and interpretation of data for the work. SM, FDR, VB, AG, CC, GR drafted the work and revised it critically for important intellectual content. All authors approved the final version to be published and acknowledged that questions related to the accuracy or integrity of any part of the work are appropriately investigated and resolved.

## Conflict of Interest Statement

The authors declare that the research was conducted in the absence of any commercial or financial relationships that could be construed as a potential conflict of interest.
